# A catalogue of putative unique transcripts from Douglas-fir (*Pseudotsuga menziesii*) based on 454 transcriptome sequencing of genetically diverse, drought stressed seedlings

**DOI:** 10.1186/1471-2164-13-673

**Published:** 2012-11-28

**Authors:** Thomas Müller, Ingo Ensminger, Karl J Schmid

**Affiliations:** 1 Department of Crop Biodiversity And Breeding Informatics, University of Hohenheim, Stuttgart, Germany; 2 Department of Biology, University of Toronto at Mississauga, Mississauga, ON, Canada; 3 Forest Research Institute of Baden-Württemberg (FVA), Freiburg i. Brsg., Germany

## Abstract

**Background:**

Douglas-fir (*Pseudotsuga menziesii*) extends over a wide range of contrasting environmental conditions, reflecting substantial local adaptation. For this reason, it is an interesting model species to study plant adaptation and the effects of global climate change such as increased temperatures and significant periods of drought on individual trees and the forest landscape in general. However, genomic data and tools for studying genetic variation in natural populations to understand the genetic and physiological mechanisms of adaptation are currently missing for Douglas-fir. This study represents a first step towards characterizing the Douglas-fir transcriptome based on 454 sequencing of twelve cDNA libraries. The libraries were constructed from needle and wood tissue of coastal and interior provenances subjected to drought stress experiments.

**Results:**

The 454 sequencing of twelve normalized cDNA libraries resulted in 3.6 million reads from which a set of 170,859 putative unique transcripts (PUTs) was assembled. Functional annotation by BLAST searches and Gene Ontology mapping showed that the composition of functional classes is very similar to other plant transcriptomes and demonstrated that a large fraction of the Douglas-fir transcriptome is tagged by the PUTs. Based on evolutionary conservation, we identified about 1,000 candidate genes related to drought stress. A total number of 187,653 single nucleotide polymorphisms (SNPs) were detected by three SNP detection tools. However, only 27,688 SNPs were identified by all three methods, indicating that SNP detection depends on the particular method used. The two alleles of about 60% of the 27,688 SNPs are segregating simultaneously in both coastal and interior provenances, which indicates a high proportion of ancestral shared polymorphisms or a high level of gene flow between these two ecologically and phenotypically different varieties.

**Conclusions:**

We established a catalogue of PUTs and large SNP database for Douglas-fir. Both will serve as a useful resource for the further characterization of the genome and transcriptome of Douglas-fir and for the analysis of genetic variation using genotyping or resequencing methods.

## Background

Douglas-fir (*Pseudotsuga menziesii* (Mirb.) Franco) is an ecologically highly variable species that occurs in two main varieties in North America. The natural range of the coastal or green Douglas-fir (*Pseudotsuga menziesii* var. *menziesii*) extends over 2,200 km from the Pacific Northwestern fog belt and the adjacent summer-dry Coastal Range and Cascade mountains to the drier coastland of Southern California. The interior or blue Douglas-fir (*Pseudotsuga menziesii* var. *glauca*) is distributed over more than 4,500 km along the dry continental climates of the montane to the subalpine Rocky Mountains from Alberta to Colorado with isolated populations reaching into Mexico. Douglas-fir grows from sea level on Vancouver Island up to 3,000 m altitude in the southern Rocky Mountains [1]. Within its natural range, Douglas-fir has evolved into a variety of genetically diverse populations adapted to contrasting ecozones (e.g. [2,3]).

Douglas-fir populations differ in their response to frost [4,5], drought [5-7], and along environmental gradients [4,8]. Like most conifer species, it is able to cope with limitations in soil water availability within its natural range [9,10]. There is a negative relationship between shoot water potential and the photosynthesis rate [6], which decreased by about 70% in water-stressed trees with a pre-dawn shoot water potential of about -1.7 MPa. In conifers such as Douglas-fir or pine, the recovery of photosynthesis upon rainfall and rewatering occurs within days together with the rapid recovery of predawn shoot water potential from stressed (around -1.5 MPa), or mildly stressed (around -1.0 MPa) to values higher than -0.5 MPa [6,11]. This high ecological, genetical and physiological diversity provides an excellent system to study the adaptation of conifer trees to contrasting environments.

Due to its rapid growth and favorable wood quality, Douglas-fir is an economically relevant species for timber production. In Europe, the area of Douglas-fir production is rising [12]. Forest practitioners appreciate the resistance of Douglas-fir against many European pathogens [13,14]. It is also expected that Douglas-fir is better adapted to future climate conditions in Central Europe than e.g. Norway spruce [15].

The Intergovernmental Panel on Climate Change (IPCC) expects increasing summer temperatures and decreasing precipitation in Central Europe in the coming years [16]. A trend towards warmer summers and more frequent summer droughts was reported in recent studies and simulations [17-19]. For this reason, it is important for forest managers to select suitable tree species or provenances that are adapted to the anticipated future climate. Currently, coastal Douglas-fir provenances are more frequently planted in Central Europe due to their superior growth performance compared to interior Douglas-firs [13,14,20]. The identification and characterization of differentially adapted provenances of coastal and interior Douglas-fir varieties has therefore high practical value.

Because of the large genome size of Douglas-firs (18.7 Gb, about 100 times the genome size of *Arabidopsis thaliana*[21,22] or 37 times the genome size of poplar [23,24]), transcriptome analysis is a cost-effective and suitable approach for the identification of candidate genes for adaptive traits and molecular markers that are linked to phenotypic variation. Transcriptomes of many species have been analyzed by next-generation sequencing technologies [25-27], and numerous coding single-nucleotide polymorphisms (SNPs) were identified in conifer species such as *Pinus contorta*, *Picea glauca* and *Pinus taeda*[26,28,29].

Douglas-firs, like other forest trees, have a high level of genetic diversity [30,31]. For example, one study identified 933 SNPs in 121 candidate genes for cold-hardiness (1 SNP per 43 bp to 1 SNP per 112 bp) in coastal Douglas-firs [32]. For this reason, transcriptome sequencing of different provenances will lead to candidate genes for differential adaptation and to many new genetic markers for the characterization of different populations.

The purpose of this study was to establish a catalogue of Douglas-fir putative unique transcripts (PUTs) enriched for drought stressed genes and to identify genetic polymorphisms as resource for further analysis such as resequencing projects, association studies, and gene expression profiling.

## Results

### Sequencing and assembly

The sequencing of twelve cDNA libraries resulted in 3,619,544 reads with an average length of 338 bp. After preprocessing, the number of reads decreased to 2,957,373. Read numbers were not equally distributed among libraries (Additional file 1). The DINM, DINS and DIWC libraries consisted of less than 200,000 reads each and the DIWM library of less than 100,000 reads (see Table 1 for an explanation of the library abbreviations). The average length of the reads decreased to 315 bp after pre-processing (Additional file 1). More than 99% of reads in each library were used for the construction of the assembly after quality trimming, with the exception of the DIWM library (95% used). A total of 2,793,051 (94.44%) reads were assembled into 141,626 isotigs (of which 275 were contigs) of at least 100 bp length. Additional file 2 contains the origin and the number of assembled reads. All isotigs were clustered in 116,311 isogroups. The mean isotig length was 623.22 bp (s.d. 437.67 bp, median: 474 bp), the mean coverage per base was 5.0 reads (s.d. 8.07), and the mean number of reads per isotig was 44.5 (s.d. 145.54). For 21,837 isotigs longer than 999 bp, the mean coverage increased to 13.66 (s.d. 11.77) reads per base. Furthermore, the mean number of reads per isotig reached 181.27 (s.d. 274.75). Length of the isotig was positively correlated with the number of reads (*r* = 0*.*4972, *P* < 0*.*0001; Additional file 3).

**Table 1:** Explanation of the cDNA library abbreviations

		**Treatment**		
**Variety**	**Tissue**	**Control**	**Mild stress**	**Severe stress**
Costal	Needles	DCNC	DCNM	DCNS
	Wood	DCWC	DCWM	DCWS
Interior	Needles	DINC	DINM	DINS
	Wood	DIWC	DIWM	DIWS

D = Douglas-fir, C/I = coastal/interior, N/W = needle/wood tissue, C/M/S = no/mild/severe drought stress.

Based on the results of the assembly, we constructed a set of PUTs as outlined in the Methods section. 42,159 of 71,392 reads with a length > 99 bp initially labelled as singletons were mapped to isotigs and were considered as false positive singletons. Therefore, the final PUT set consisted of 170,859 sequences (141,626 isotigs and 29,233 singletons) with an average sequence length of 564.6 bp (s.d. 420.86 bp, median: 431 bp, Additional file 4). As no reference sequence of Douglas-fir was available, we used the PUT set as reference for the following analysis, including functional annotation and SNP detection.

### Functional annotation of the PUTs

**Figure 1:**
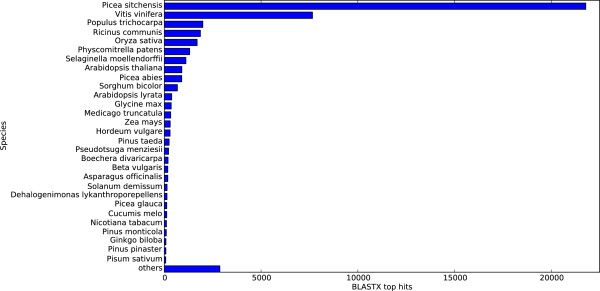
**Distribution of the top hits species of the BLASTX search of the PUTs from the assembly against NCBI’s*nr* database.**

**Figure 2:**
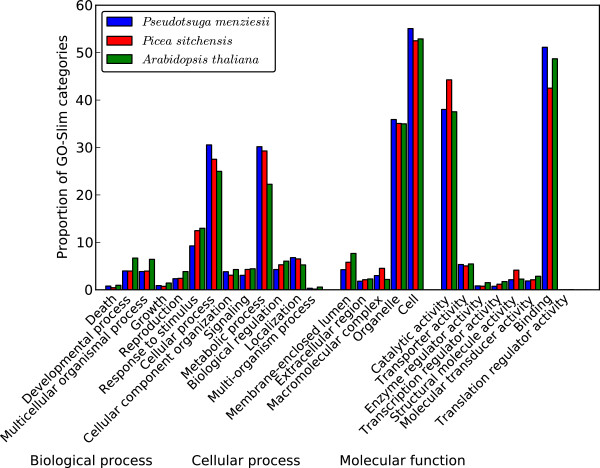
**Comparison of the GO-Slim categories.** Comparison of the distribution of the GO-Slim categories of the Douglas-fir PUT set versus *Picea sitchensis* and *Arabidopsis thaliana* at GO level 2. Transcriptome data of *P. sitchensis* and *A. thaliana* were obtained from NCBI and TAIR databases, respectively.

For functional annotation, we compared all PUTs to the NCBI *nr* database using BLASTX with an e-value cutoff of *e*^−10^. At least one BLAST hit was obtained with 46,645 transcripts. If only the best hit of each transcript is considered, a total of 20,604 different sequences (unique hits) were hit in the *nr* database. The largest number of hits was against *Picea sitchensis* sequences, followed by *Vitis vinifera* (Figure 1). In the subsequent analysis, Blast2GO assigned at least one GO term to 39,624 transcripts. For the three main GO categories, 34,660 transcripts were assigned a GO term from the molecular function category, 28,714 from the biological process, and 24,166 from the cellular component category. To compare the distribution of GO terms of the Douglas-fir transcriptome with the distribution of GO terms of transcriptomes from other species, we also applied Blast2GO to the *Arabidopsis thaliana* and *Picea sitchensis* sequences downloaded from TAIR and NCBI, respectively. We chose these two species for comparison because *A. thaliana* is a well studied model species with a well studied transcriptome and *P. sitchensis* is the species with most top BLASTX hits in our Douglas-fir PUT set. Figure 2 and Additional file 5 show that the distributions of GO terms at GO level 2 to 5 for each of the three ontology classes are highly similar for all three species.

### Identification of treatment-specific PUTs

**Figure 3:**
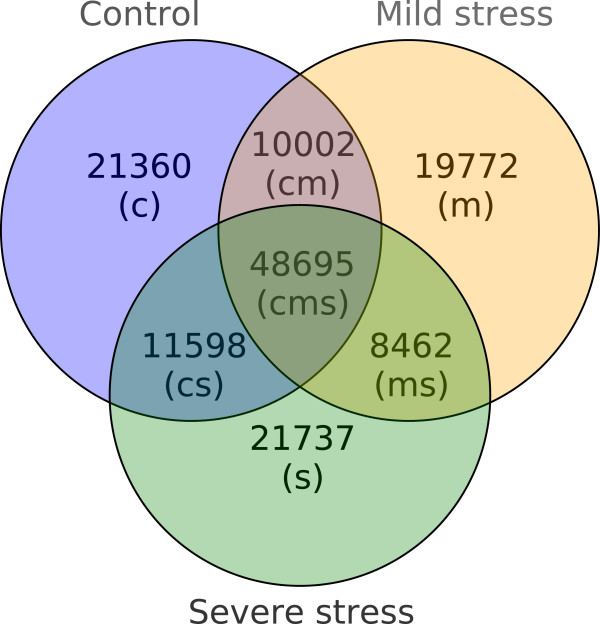
**Composition of isotigs.** Venn diagram showing the number of non-singleton PUTs (i.e. isotigs) consisting of reads from (one or several) libraries of one or more treatment(s). E.g. 21,737 isotigs are composed of reads originating from one or several of the severe stress libraries (DCNS, DINS, DCWS, DIWS). *c* = control, *m* = mild stress, *s* = severe stress.

The isotigs (non-singleton transcripts of the PUT set) were divided and clustered according to the origin of their reads (Figure 3). About one third (34.38%) of the isotigs contained reads from all three treatments and were therefore grouped in the *cms* group. The average length of isotigs in the *cms* group was 995.32 bp (Table 2). Each of the *cm*, *cs*, and *ms* groups contained 6-8% of the isotigs with an average isotig length of 489 to 496 bp. The *c*, *m*, and *s* groups contained 14-15% of the isotigs in each case. The average lengths of those isotigs were between 393 and 405 bp. The search for specific keywords in the BLASTX results revealed that 1,503 different isotigs coming from 998 isogroups had a BLASTX hit containing one of the keywords related to stress (Table 3, Additional file 6, Additional file 7). 134 of those isotigs coming from 132 isogroups were part of the *m*, *s*, or *ms* groups and will serve as top candidate genes in future studies. We expected that *cms* group sequences are more conserved than sequences assigned to the remaining groups because drought stress specific sequences may evolve faster or are of a more recent evolutionary origin than common or widely expressed genes.

**Table 2:** Percentages of isotigs with BLASTX hits

			**% of isotigs with BLASTX hits vs.**		
	**Avg. lengthof isotigs**	**% of all isotigs**	***nr***	***ara***	***picea***
*c*	393.33	15.08	16.29	9.11	10.59
*m*	392.77	13.96	14.13	6.22	7.62
*s*	405.5	15.35	14.77	7.32	8.75
*cm*	488.89	7.06	24.59	15.8	16.12
*cs*	493.72	8.19	25.38	17.44	17.19
*ms*	496.32	5.97	19.14	11.05	11.18
*cms*	995.32	34.38	58.11	47.24	46.06

Percentages of isotigs (non-singleton PUTs) with a BLASTX hit against the *nr*, *ara*, and *picea* databases within the groups (see Figure 3). *c* = control, *m* = mild stress, *s* = severe stress, *cm* = control and mild stress, *cs* = control and severe stress, *ms* = mild and severe stress, *cms* = control, mild and severe stress.

**Table 3:** Keyword search in BLASTX results

	**Isotig group**						
**Search term**	***c***	***m***	***s***	***cm***	***cs***	***ms***	***cms***
”Drought”	4	3	8	5	13	3	103
”Water-deficit”	0	0	4	0	5	0	29
”Water-stress”	6	2	6	4	6	1	109
”Osmotic-stress”	4	1	6	2	7	2	58
”Heat-stress”	2	0	1	0	2	0	27
”Heat-shock”	24	17	31	21	23	15	466
”Dehydration”	20	7	17	14	18	1	205
”Abscisic acid”	7	1	8	5	10	2	142
”ABA-responsive”^1^	0	0	2	1	1	1	25
”ABA-induced”	1	0	2	2	1	0	27
”ABA receptor”	0	0	2	0	0	0	20
”Pyrabactinresistance 1”	0	0	2	0	0	0	10
”Snf1-relatedprotein kinases”^2^	4	2	2	2	3	7	69
”DREB1”^3^	2	0	0	1	1	0	9
”DREB2”	2	0	0	2	2	0	14
”C-repeat binding”	0	0	0	1	1	0	4
”ERD”^4^	7	4	9	3	8	2	112
”CIPK”^5^	2	3	2	0	2	6	47
”CDPK”^6^	0	2	1	7	1	0	39
”CBL1” ^7^	5	1	0	3	6	1	72
”PKS3”^8^	0	2	0	0	2	0	12
Different isotigs	66	33	69	58	71	32	1,174

Number of isotigs (non-singleton PUTs) with a BLASTX hit containing a keyword for each group (see Figure 3). *c* = control, *m* = mild stress, *s* = severe stress, *cm* = control and mild stress, *cs* = control and severe stress, *ms* = mild and severe stress, *cms* = control, mild and severe stress.

^1^ABA = abscisic acid.

^2^Snf = sucrose non-fermenting.

^3^DREB = dehydration-responsive element-binding.

^4^ERD = early responsive to dehydration.

^5^CIPK = CBL-interacting protein kinase.

^6^CDPK = calcium-dependent protein kinase.

^7^CBL = calcineurin B-like protein.

^8^PKS = phytochrome kinase substrate.

To test this hypothesis, we determined the proportion of significant BLASTX hits within each group of isotigs against the *nr*, the *ara*, and the *picea* databases (Table 2). Most hits were observed in the *cms* group (e.g. 58.11% against *nr*) and the least number of hits in the *m* group (14.13% against *nr*). However, there is a highly significant correlation between the average length of isotigs and percent BLAST hits (e.g. hits against *ara*, *P* < 0*.*0001, Table 4), and also between the total sequence length of each isotig group with the proportion of BLAST hits (e.g. hits against *ara*, *P* = 0*.*003). Hence, the differences in the proportion of BLAST hits among classes of isotigs are not a result of differential evolutionary conservation, but of the amount of sequence data in each class.

**Table 4:** BLASTX and Blast2GO results divided by isotig length

	**All PUTs**	**< 501**	**501 – 1,000**	**1,001 – 1,999**	**> 2,000**
Number of PUTs	170,859	106,296	42,760	19,589	2,214
Total sequence [Mbp]	96.5	35	26.4	29.7	5.4
Hits with *nr*	27.3%	13.5%	36.9%	75.5%	87.8%
Hits with *ara*	19.6%	7.9%	25.1%	64.4%	82.6%
Hits with *picea*	19.6%	8.5%	26.3%	58.7%	71.4%
Isotigs with assigned GO term	23.2%	11.9%	26.6%	63.9%	82%

Results of similarity searches with BLASTX and functional annotation using Blast2GO subdivided by transcript length in bp.

### SNP identification

**Figure 4:**
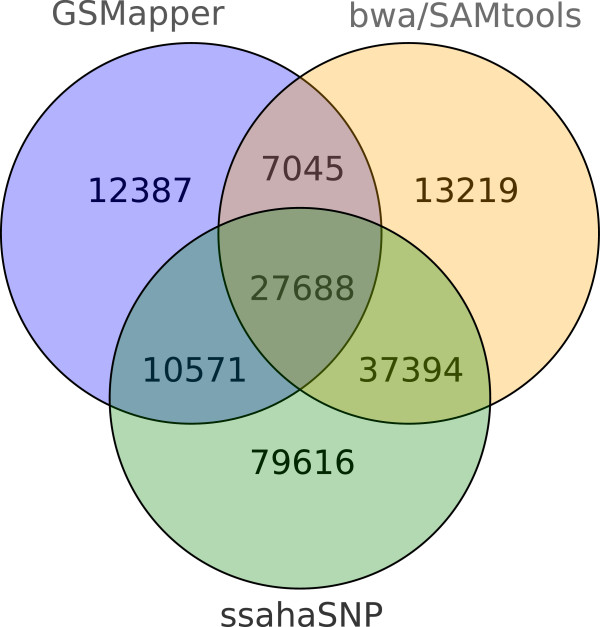
**Number of SNPs.** Number of SNPs identified by the SNP detection tools GSMapper, ssahaSNP, and bwa/SAMtools. 27,688 SNPs were detected by all three tools and are considered to be the most reliable SNPs.

SNP detection was performed with three different programs, GSMapper, ssahaSNP, and bwa/SAMtools, to minimize the number of false positives. PUTs obtained from the assembly served as reference. The programs detected 57,691 (Newbler), 155,269 (ssahaSNP), and 85,346 (bwa/SAMtools) SNPs, resulting in a total number of 187,653 different SNPs. However, only 27,688 SNPs were detected by all three tools (Figure 4). These SNPs were selected for further analysis because we consider them as most reliable true positive polymorphisms. These SNPs were distributed over 10,517 different PUTs of 10,054 different isogroups. Most transcripts harbored only a single SNP and 2,499 transcripts contained more than three SNPs. A total of 23 SNPs were detected in the most polymorphic PUT. In the 7,684 transcripts with at least one SNP and a significant match against the *nr* database, 5,378 SNPs were classified as synonymous and 4,129 as non-synonymous.

In addition, we estimated the polymorphism level of the transcriptome by dividing the number of SNPs with the total number of different nucleotides in PUTs (as the same contig can contribute to several isotigs, see Additional file 8). If only the most reliable SNPs are considered, the estimated nucleotide diversity (0.04%, corresponding to approximately 1 SNP per 2,530 nucleotides) is very low. Using all SNPs identified by Newbler, bwa/SAMtools and ssahaSNP separately, resulted in estimated polymorphism levels of 0.08% (1 SNP per 1213 bp), 0.12% (1 SNP per 820 bp), and 0.22% (1 SNP per 451 bp), respectively.

To investigate differences in the level of genetic diversity between coastal and interior Douglas-firs, we divided the SNPs into groups depending on whether their alleles segregated in coastal or interior provenances, or in both (Table 5). The majority of SNPs are polymorphic in both coastal and interior provenances (Table 6), but coastal provenances have a higher number of provenance-specific alleles, as seen in the comparison of ci/c (7,158 SNPs) versus ci/i (2,547 SNPs) groups.

**Table 5:** Groups of SNPs

Origin of reads confirming the reference nucleotide	c	i	c	i	c	ci	i	ci	ci	
Origin of reads confirming the variant nucleotide	c	i	i	c	ci	c	ci	i	ci	
Group name	c/c	i/i	c/i		ci/c		ci/i		ci/ci	

Partitioning of SNPs into groups depending on the origin (coastal vs. interior) of sequence reads. c: only reads of coastal libraries; i: only reads of interior libraries, ci: reads of coastal as well as interior libraries; ci/ci: both possible nucleotides were confirmed by reads of coastal and interior libraries; c/i: one of the possible nucleotides at the SNP position was confirmed only by reads of coastal libraries, the other nucleotide only by reads of interior libraries; etc.

**Table 6:** Summarized number of SNPs

**Origin of reads at SNP position**	**Number of SNPs**
ci/ci	15,843
ci/c	7,158
ci/i	2,547
c/i	886
c/c	817
i/i	437

Number of SNPs with a specific composition of reads. ci/ci: variant and reference nucleotide appeared in reads from coastal and interior libraries; ci/c, ci/i: variant or reference nucleotide appeared only in reads of the coastal or interior libraries, the other one appeared in reads of both kind of libraries; c/i: variant or reference nucleotide appeared only in reads of the coastal libraries, the other one only in reads of the interior libraries; c/c, i/i: variant and reference nucleotides appeared only in reads of coastal or interior libraries.

## Discussion

### Sequencing and assembly

Next generation sequencing (NGS) has now a major impact on the genome-wide analysis of transcriptomes in non-model species [25-27]. To achieve a comprehensive characterization of the protein-coding genome of Douglas-fir, we exposed young seedlings from different provenances to drought stress treatments and generated normalized cDNA libraries to enrich for rare transcripts or genes not constitutively expressed. All libraries were assembled into a single assembly to maximize the number of reads per transcript and to improve the quality of assembly and SNP annotation. There is a strong relationship between the number of reads and the length of a transcript, confirming the observation that longer transcripts consist of more reads than shorter transcripts [33]. The number and average length of the reads of four libraries (DINM, DINS, DIWC, and DIWM) were below expectation [26,34,35], probably because of problems during the sequencing process. However, we did not exclude these libraries, because they contributed the same proportion of reads to the assembly as the other libraries (> 95% of the reads of each library).

94.44% of all reads were assembled into isotigs during assembly, which is a high proportion compared to similar 454 transcriptome assemblies. For example, 88% of reads were assembled in *Melitaea cinxia*[36] and *Eucalyptus grandis*[25], 78% in *Pandinus imperator*[37], and 48% in *Pinus contorta*[26]. One cause for the high proportion in our data is the stringent preprocessing of reads, which excluded most uninformative reads prior to the assembly. The number of PUTs in the assembly (170,859) exceeds the number of expected genes in conifer genomes, which ranges from 30.000 to 50.000 genes [38]. Nevertheless, the PUT set is smaller than the one obtained with *Pinus contorta* (303,450 transcripts) [26], but larger than in other 454 transcriptome sequencing projects (e.g. 44,469 transcripts in waterhemp [39], 108,297 transcripts in a butterfly species [36]). It should be noted that it is difficult to compare numbers of transcripts in different projects, because they are influenced by the genome and transcriptome sizes of the sequenced organism, the assembly method used [34], and the number of reads used for the assembly. Overall, the high number of transcripts compared to the expected number of genes is likely a result of incompletely assembled genes.

The average length of transcripts is 564.60 bp (median 431 bp, Additional file 4), which is approximately half of the expected average gene length in eukaryotes (1346 bp) [40].

### Functional annotation of PUTs

We annotated the PUTs by using stringent BLASTX searches against the *nr* database from NCBI with a cutoff e-value of *e*^−10^. Assuming that each unique blast hit represents a different transcribed gene, we tagged 20,604 genes of the Douglas-fir genome. This number is similar to other projects in *Pinus contorta* with 17,321 tagged genes [26] and is lower than the estimated total number of about 30,000 transcribed genes in white spruce *Picea glauca*[38]. If we further assume that the number of unique blast hits equals the number of transcribed genes and that the transcriptome sizes of Douglas-fir and white spruce are comparable, it seems that the PUT set generated in this study tags about two thirds of the protein-coding genes of Douglas-fir. The missing third likely comprise (1) cDNAs that were excluded from assembly because of low quality; (2) genes that are expressed at different developmental stages, growth conditions, or tissues and were thus not represented in the twelve cDNA libraries despite the normalization process; and (3) non-conserved genes, which are either lineage-specific and not yet present in the *nr* database or rapidly evolving genes with e-values > *e*^−10^ in the BLASTX comparisons. Since about 75% of the transcripts do not result in significant BLASTX hits, one may speculate that a large proportion represents non-conserved genes.

Gene Ontologies (GOs) provide a standardized set of terms to describe genes and gene products consistently in different species and databases [41]. GO terms are widely used for annotation and for comparisons of gene products of different species (e.g. [26,42]). The similarity of the GO annotation distributions of Douglas-fir PUTs to the well-characterized transcriptome of *A. thaliana* and the protein sequences of *P. sitchensis* (Figure 2) suggests that the PUT set broadly represents the Douglas-fir transcriptome and can be viewed as being representative for further applications and investigations.

### Identification of drought stress related genes

Dividing PUTs consisting of multiple reads (i.e. the isotigs) by the origin of their reads is a simple, but useful method to identify potential treatment-specific sequences. About 50% of isotigs consist of reads from the single treatment groups *c*, *m*, or *s*. On average, they are shorter than isotigs containing reads from at least two different treatments. The isotigs of the *m* and *s* groups, but also the *ms* group, were considered to be top candidates for drought stress tolerance or resistance. However, since most treatment-specific isotigs consist of only two or three reads that originated from a total of twelve cDNA libraries, we reasoned that the power of a statistical model to detect significant differences is low. Therefore, we compared the extent of evolutionary conservation between groups as judged by the proportion of significant BLAST hits. This analysis is based on the notion that widely expressed genes are under stronger selective constraint than treatment-specific genes [43,44]. Under the assumption that constitutively expressed genes are expressed in all different treatments, we expected that *cms* group isotigs are more conserved than isotigs from the *c*, *m*, and *s* groups. Since the libraries were normalized and cDNA levels do not represent true expression levels, we restricted our analysis to presence-absence patterns. The differences in the fractions of BLASTX hits in single treatment groups and the *cms* group suggested that genes expressed in all three treatments are more conserved. However, if groups are corrected for the total amount of sequence data, *cms* group isotigs are not more conserved than treatment-specific isotigs, because the main determinant for a BLAST hit is isotig length which is longer in *cms* isotigs (Table 4). This pattern was also observed in white spruce [38].

In addition to testing the general hypothesis that treatment-specific genes are less conserved than widely-expressed genes, we also parsed BLASTX results for drought stress related keywords to find potential drought stress related PUTs. We expect that the 1,503 transcripts with a BLASTX hit containing one of the keywords are probably involved in the Douglas-firs response to drought (Table 3). More than 1,100 candidate PUTs are part of the *cms* group and only 134 candidates are part of the *m*, *s*, and *ms* groups. This reflects that the response to drought seems to be mainly facilitated through changes in gene expression levels via up- or down-regulation. The small set of 134 PUTs exclusively induced by drought stress appears to contribute to a specific drought response, but this needs to be further verified because their expression pattern may reflect a sampling artifact. Even though the function of those PUTs may not be conserved across large evolutionary distances, the identified PUTs serve as top candidates for further analysis of sequence and expression variation in comparisons of differentially adapted (e.g. coastal and interior) Douglas-fir provenances.

### Analysis of genetic variation

The construction of the cDNA libraries representing genetically different provenances allowed the detection of SNPs for later analysis of patterns of genetic variation. The two most important results are the high proportion of shared polymorphisms and the strong influence of the SNP calling algorithm on the number of detected SNPs. By taking a conservative approach and considering only those SNPs that were called by all three programs, only 27,688 (highly reliable) SNPs were obtained, which is only about half of the number detected with gsMapper, which identified the lowest number of SNPs (57,691). Since the numbers of called SNPs differed highly between SNP detection tools, our results indicate that SNP calling from next generation sequencing data depend to a high degree on the software tools used. Therefore, results should be interpreted with caution, if relying on a single SNP detection approach only. To our knowledge there are no systematic studies yet that compared the accuracy of different SNP callers with next-generation sequencing data.

A comparison of the SNP density (SNPs per kb) of the most reliable SNPs with published data shows that the former is an underestimate of the true level of sequence variation in Douglas-fir. The SNP density is 1 SNP for every 2,530 bp, whereas other studies estimated an average SNP density from 1 SNP per 43 bp to 1 SNP per 112 bp using Sanger sequencing protocols [32]. The reasons for the large difference to the reported SNP density are probably the stringency criteria used and the better quality of base-calling using Sanger sequencing. If we take only the SNPs identified by bwa/SAMtools or ssahaSNP in account, the SNP density increases to 1 SNP per 820 bp and 1 SNP per 451 bp, respectively.

Nevertheless, our sequence data make a significant contribution to the number of Douglas-fir SNPs available for further applications. Until now, only around 1,300 SNPs have been identified in Douglas-fir [32,45]. If only the most reliable SNPs are considered, a key result is the large number of SNPs whose alleles are segregating in both the coastal and interior provenances (15,483 SNPs, ci/ci category in Table 6). In only 5% of SNPs (886, c/i) the two alleles are specific to coastal and interior provenances, respectively. This high proportion of shared polymorphisms indicates either a high level of shared ancestral polymorphisms between the two main Douglas-fir varieties, or recent, possibly pollen-mediated gene flow. The comparison of SNPs, in which only one of the two alleles is shared between coastal and interior provenances suggest a higher level of genetic diversity in coastal provenances because three times as many SNPs are polymorphic for both alleles in the coastal (7,158 SNPs in the ci/c group) than in the interior accessions (2,547 SNPs in the ci/i group). This difference is also observed for SNPs which were called only in either the interior or coastal provenances because no reads were available from the other provenance, respectively (817 SNPs in the c/c *versus* 473 SNPs in the i/i group). Although these results are consistent with earlier studies on the genetic diversity of Douglas-fir varieties [46,47], they are also certainly influenced by the different numbers of reads originating from coastal and interior cDNA libraries (1,757,542 vs. 1,076,192). Since there are 70% more reads from the coastal provenances, the probability of finding a polymorphism in these provenances is increased and needs to be accounted for in further conclusions.

Different numbers of reads can be accounted for by using methods for population genetic inference developed for next-generation sequencing that account for differences in read numbers from individuals or pools of individuals in estimating allele frequencies and population parameters [48,49]. However, such an approach does not work in the present study because allele frequencies depend on the sampled individuals in a library, the gene expression level and the effect of normalization on read numbers. Unbiased population genetic estimators like Tajima’s *Π* can be calculated from 454 data [50], but as the coverage at most SNP positions is much smaller than the total number of individuals, the results are not reliable. The development of genotyping and resequencing arrays using the present set of PUTs to estimate SNP allele frequencies and population genetic inference will allow accurate and unbiased estimates of nucleotide diversity.

## Conclusions

In this study we established a catalogue of Douglas-fir putative unique transcripts (PUTs) enriched for drought stress induced genes. Although the real magnitude of the transcriptome is yet unknown, we estimate that the majority of the transcriptome has been tagged by the PUT set presented here. This is based on the results of the functional annotation and the comparison of the GO term distributions with those of *Arabidopsis thaliana* and *Picea sitchensis*. By analyzing sequence variation in the PUTs we detected thousands of new SNPs. Furthermore, we identified drought stress specific candidate sequences. Taken together these data represent a useful resource for the next steps in the characterization of the Douglas-fir genome and transcriptome and the association of genetic variation with phenotypic traits such as adaptation to different ecogeographic environments.

## Methods

### Plant material and library preparation

1.5 year old Douglas-fir seedlings were obtained from tree nurseries in British Colombia (Canada), Washington, Colorado, and New Mexico (USA) and grown in the greenhouse in a mixture of soil:perlit:sand (50:25:25). All seedlings were fertilized with Osmocote Exact Hi End 5-6m (Scotts International BV, Heerlen, NL). Potted seedlings were watered every second day. Drought stress experiments started after two month of growth in the greenhouse, when visual inspection of the seedlings indicated a well developed root system. For the experiments, seedlings were randomly assigned to one of three different treatments: (1) control seedlings kept under well watered conditions, (2) mildly water stressed seedlings (predawn water potential between -0.7 and -1.0 MPa) and (3) severely water stressed seedlings (predawn water potential between -1.5 and -2.0 MPa). Water stress was imposed by withholding watering until a desired water potential had been reached [11]. Water potential was assessed by repeated measurements of predawn needle water potential using a Scholander pressure chamber to assess the level of water stress [51]. Within about 3-4 and 5-6 weeks, the target water potential was observed in the mildly and severely water stressed seedlings, respectively, and needles and sections of the stem (wood tissue) were harvested. Tissue from control seedlings was harvested in parallel in order to obtain samples from similarly aged plant material. Tissue samples were immediately frozen in liquid nitrogen and stored at -80°C for later extraction of RNA.

Frozen needles or sections of the stem were homogenized using mortars and pistils chilled with liquid nitrogen until a fine powder was obtained. Total RNA was extracted from 300 mg aliquots of the frozen powder using the CTAB method [52]. Isolated RNA from individual seedlings was then quality checked using Qiaxcel (Qiagen, Hilden, Germany).

Aliquots of the RNA from several seedlings and several provenances were then combined for synthesis into a total of twelve pooled RNA samples. Six of these pooled samples represented a subset of coastal and six samples represented a subset of interior Douglas-fir. Each of these two subsets included two sets of pooled RNA samples from either needle tissue or from wood tissue. Finally, each of these tissue specific sets consisted of one pooled RNA sample from control, mildly water stressed or severely water stressed seedlings (Table 1, Additional file 9).

Normalized cDNA libraries were generated by Evrogen LAB (Moscow, Russia). Starting from 0.3 Âµg of total RNA double-stranded cDNA was synthesized using SMART Oligo II oligonucleotides and CDS primers (SMART Oligo II 5’ –AAGCAGTGGTATCAACGCAGAGTACGCrGrGrG– 3’, CDS primer 5’ – AAGCAGTGGTATCAACGCAGAGTA-d(T)30– 3’) [53]. Amplified cDNA was then purified using the QIAquick PCR purification kit (Qiagen, CA, USA), concentrated by ethanol precipitation and then diluted to a final cDNA concentration of 50 ng/Âµl. SMART amplified cDNAs were then normalized [54]. Normalization included a cDNA denaturation/reassociation step followed by treatment with duplex-specific nuclease (DSN, [55]) and subsequent amplification of the normalized fraction by PCR using SMART PCR primers (SMART PCR primer 5’ –AAGCAGTGGTATCAACGCAGAGT– 3’).

454 sequencing of the normalized cDNA libraries was carried out by Seq-IT (Kaiserslautern, Germany) using a Genome Analyzer FLX with 454 titanium chemistry (Roche, Basel, Switzerland). Prior to sequencing, each cDNA library was first fragmented. Fragments were tagged with multiplex identifiers (MIDs) to allow library identification of the reads obtained from parallel sequencing of the libraries on the Genome Analyzer FLX. In total three titanium runs were performed, with 1.5 runs analyzing the needle libraries, and 1.5 runs analyzing the wood libraries. The proprietary genome analyzer software was used for the first preprocessing of sequence reads including the assignment of quality scores to generate.sff-files for further processing and assembly of the data.

### Preprocessing

The resulting.sff-files were extracted with the sff_extract tool [56]. All sequences with at least one ’N’ were removed. The preprocessed files were used as input for SnoWhite (release 1.1.3) [57], a cleaning pipeline for cDNA sequences that uses SeqClean [58] and trims polyA/T. All sequences shorter than 50 bp or with a polyA/T repeat of at least 8bp at either end were discarded. The longer part of the sequence was retained if internal polyA/T tracts were detected. As the assembly program operates in flowgram signal space it is recommended to use.sff-files as input. Thus, the original.sff-files were altered according to the changes made during the preprocessing steps using custom Python scripts. Those altered.sff-files were loaded into the assembler.

### Assembly and mapping

Sequences were assembled with Newbler v2.6 using default parameters supplemented by the -cdna and -urt options [34,59]. Newbler constructs a set of contigs (contiguous sequences), representing assembled reads. Unassembled reads were marked as singletons, repeats, outlier (e.g. chimeric reads), or too short. Isotigs consist of contigs connected by a subset of reads (Additional file 8). An isogroup is a group of different isotigs of the same multiple alignment. Isogroups represent genes, isotigs correspond to alternatively spliced transcripts, and contigs correspond to exons. This is a simplified view because contigs and isotigs can also contain sequences of untranslated regions. Independent contigs that were not part of an isotig were simply considered as isotigs to facilitate the analysis.

All twelve libraries were assembled together. Based on the assembly, we created a set of PUTs. We first searched for false positive singletons, i.e. reads that were marked as singletons although they matched nearly perfect to an existing isotig. For this purpose, all reads marked as singletons were mapped to the isotigs of the assembly using ssaha2 [60] with default parameter settings. Reads were mapped only if the pairwise sequence identity with a reference isotig was at least 98% of the alignment length. Unmapped reads were considered as real singletons and checked for duplicates. The final PUT set consisted of the isotigs and the singletons of the assembly representing all different transcripts found in the dataset. In particular, i.e. PUTs can be the only possible transcript of a gene, only a part of a longer transcript that can not be found within the data, alternatively spliced variants of a gene, but also the product of misassemblies. Sequences shorter than 100 bp were excluded to dismiss potentially uninformative sequences.

### SNP detection

**Figure 5:**
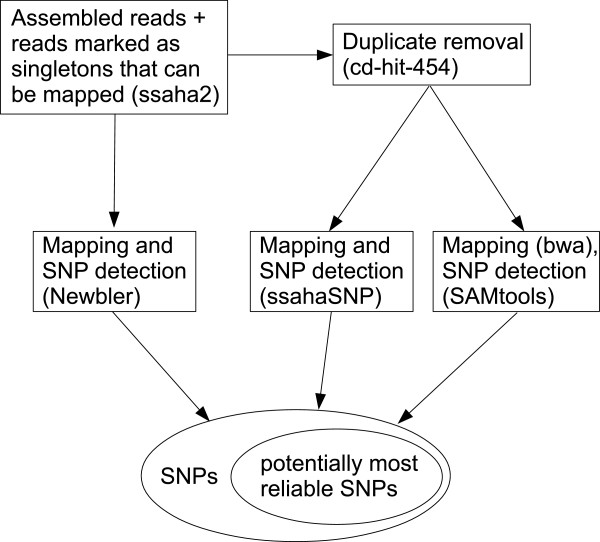
**Workflow of the SNP detection.** All reads that were assembled and all reads that were marked as singletons but that can be mapped using ssaha2 (false positive singletons) served as input. Mapping of the reads and SNP detection was performed by three programs: Newbler, ssahaSNP, and bwa/SAMtools. For the latter two, the duplicates were removed using cd-hit-454. The workflow resulted in a set of SNPs, whereby those found by all tools are probable the most reliable SNPs.

SNPs were identified with GSMapper [59], ssahaSNP [60], and bwa/SAMtools [61,62]. Each program detected a different number of SNPs. Therefore, we combined the results of the three programs and considered the SNPs identified by all three tools as a set of potentially most reliable SNPs (Figure 5). We used the sequences of the PUTs derived from the assembly as reference for the SNP detection. To avoid sequencing errors from being considered as SNPs, we required for each tool that the reference nucleotide as well as the variant nucleotide were confirmed by at least three reads each. Hence, the minimum coverage per SNP position was six reads. 

GSMapper v2.6 was run with default parameters for cDNA libraries. We constructed a.sff-file including all assembled reads of the assembly and all singleton reads that were mapped to the isotigs with ssaha2 (false positive singletons). All reads of that.sff-file were mapped against the reference sequences with GSMapper. The resulting file was parsed for SNPs using a custom script.

ssahaSNP v2.5.3 does not handle duplicate reads internally like GSMapper. Therefore, duplicate sequences were removed from the set of all assembled reads and all false positive singletons using cd-hit-454 v3.1.2 [63]. A.fastq-file was produced using the corresponding fasta and quality files of the non-duplicate sequences. ssahaSNP was run with default parameters and mapped the reads of the.fastq-file against the PUTs. The results were further processed using the parse_SNP tool provided within the ssahaSNP package and custom scripts to extract SNPs that matched our criteria.

The third approach combined two tools, bwa v0.5.9 for mapping and SAMtools v0.1.16 for variant calling. The same.fastq-file that was used for ssahaSNP was used as input for bwa. As recommended for 454 reads, the bwasw option of bwa was used. The reads were mapped against the sequences of the PUTs. SAMtools was applied to convert the resulting.sam-file to a sorted.bam-file and to call the variants in that.bam-file. The resulting SNPs were again parsed to report only those SNPs with at least three reads confirming the variant and at least three reads confirming the reference nucleotide. The final set of potentially most reliable SNPs was obtained by combining the results of the three approaches and extracting those SNPs that were detected by all three programs.

Synonymous and non-synonymous polymorphisms were detected using the results of the BLASTX search of the assembly against NCBI’s non-redundant *nr* database (see below). All high-scoring segment pairs (HSP) of the top hit of each query were considered. Using the information of the BLAST results, we examined whether a SNP was in a coding or non-coding region of a gene. For SNPs in coding regions, we determined the amino acid at the corresponding position with the reference nucleotide as well as with the variant nucleotide to call synonymous or non-synonymous SNPs.

According to the criteria for SNP detection, a transcript was covered by at least six reads at each SNP position and at least three reads had to confirm each allele of a SNP. Each of the two alleles of a biallelic SNP can therefore include reads from coastal or interior varieties only, or from both varieties, resulting in nine combinations, which are summarized in Table 5. Since there is no reference genome sequence of Douglas-fir available, it was not possible to decide which of the two nucleotides was the reference or the variant nucleotide. Therefore, we pooled some combinations to compare the results independently of the classification of a nucleotide as reference or variant in our results.

### BLAST searches and annotation

To investigate the evolutionary conservation of the transcripts, we constructed two databases: one containing *Picea sitchensis* protein sequences downloaded from the NCBI data repository (*picea* database [64]) and one containing *Arabidopsis thaliana* sequences downloaded from TAIR (*ara* database [65]). The *picea* database consisted of 18,816 and the *ara* database of 35,381 sequences. PUTs were blasted against those two databases as well as against NCBI’s non-redundant *nr* database using BLASTX v2.2.25+ with an e-value threshold of *e*^−10^.

Results of the BLASTX search of PUTs against *nr* database were used as input for Blast2GO v2.4.9 [66]. Blast2GO was utilized for the functional annotation with gene ontology (GO) terms. The first step in Blast2GO was the mapping, in which GO terms associated with the hits obtained during the BLASTX search were retrieved. In the annotation step, functional terms were assigned to the sequences based on the retrieved set of GO terms per sequence using Blast2GO’s annotation score. Furthermore, we used a local version of InterProScan [67] (version 4.8) to search protein signatures in the InterPro database [68]. With the local version it was possible to analyze nucleotide sequences in all six possible open reading frames. Due to the long running time of some of the InterProScan applications, we used only a subset of them that included blastprodom, fprintscan, hmmpfam, hmmpanther, hmmtigr, hmmsmart, patternscan, and seg [68]. The results of the InterProScan were imported into Blast2GO to improve annotations. Annotations were further refined using Annex and GO-Slim, both of which were available within Blast2GO [69,70]. Annex augments annotations by finding relationships between different GO terms and adding implicit annotations. GO-Slim represents a reduced set of GO terms that gives a useful summary of the all GO terms. Blast2GO provides organism specific GO-Slim mappings of which the plant specific mapping was chosen. For a better comparison of GO terms, functional annotations were generated for the protein sequences of *P. sitchensis* and *A. thaliana* used in the *picea* and *ara* databases. A BLASTP (v2.2.25+) search with an e-value of *e*^−5^ against NCBI’s non-redundant protein sequences was done before running Blast2GO. We did not annotate these two data sets with InterProScan, but with Annex and GO-Slim. The results of functional annotation of PUTs were compared to the results of the functional annotation of *P. sitchensis* and *A. thaliana*.

### Identification of drought stress related genes

Two approaches were used to identify potential drought stress related genes. In the first approach, we divided the non-singleton PUTs, i.e. the isotigs, of the assembly by the origin of their reads into seven groups. The groups were named according to the libraries from which the reads were derived (*c*, *m*, *s*, *cm*, *cs*, *ms*, *cms*, where *c* stands for control, *m* for mild stress, and *s* for severe stress, *cm* for control and mild stress etc.). The isotigs in the *c*, *m*, and *s* groups were assumed to contain most likely treatment- specific sequences, as they contained isotigs composed of only reads of one treatment. Therefore, we expected to find drought stress related sequences mainly in the *m* and *s*, but also in the *ms* groups. For the second approach, the BLASTX results were searched for specific keywords to identify candidate genes previously assigned to drought, water stress, or other stress related pathways (Table 3) [71-73].

### Data availability

The sequence reads were submitted to the ENA Sequence Read Archive (SRA) under study accession number ERP001358 (http://www.ebi.ac.uk/ena/data/view/ERP001358). PUTs, annotated SNPs, and Blast2GO results will be available from http://www.treeversity.org. BLASTX results and Python scripts used for the analysis are available upon request.

## Abbreviations

SNP: Single nucleotide polymorphism.

## Competing interests

The authors declare that they have no competing interests

## Authors’ contributions

IE designed, conducted and coordinated greenhouse and experimental work. IE and KS designed the sequencing experiment. TM analyzed the sequence data. TM, IE and KS wrote the manuscript. All authors read and approved the final manuscript.

## Supplementary Material

Additional file 1 **Characteristics of the libraries.** Number of reads and average read length per library before and after the pre-processing steps. Click here for file

Additional file 2 **Read composition of the assembly.** The origin as well as the number of reads assembled or otherwise marked by Newbler is illustrated. Click here for file

Additional file 3 **Log-log plot of assembled reads versus the sequence length.** The log-log plot shows that the sequence length is depending on the number of reads assembled to the sequence. Click here for file

Additional file 4 **Number of isotigs per sequence length.** Number of isotigs per sequence length. Reads of all twelve cDNA libraries were assembled using Newbler. Click here for file

Additional file 5 **Comparison of the GO-Slim categories level 3 - 5.** Comparison of the distribution of the GO-Slim categories of the Douglas-fir PUTs set versus *Picea sitchensis* and *Arabidopsis thaliana* at GO level 3 to 5. Transcriptome data of *P. sitchensis* and *A. thaliana* were obtained from NCBI and TAIR databases, respectively (See text for details). Click here for file

Additional file 6 **Number of identical BLASTX hits of different combination of groups after the keyword search.** This table lists the number of identical BLASTX hits of different combination of groups after the keyword search. If combinations of sets are not listed, there were no equal BLASTX hits. Click here for file

Additional file 7 **BLASTX keyword search results.** This file lists in a tab separated style for each BLASTX keyword search hit the following informations: keyword, isotig id, isotig group, hit id, hit definition, e-value. If there were more than one hit per keyword and isotig, only the best hit (i.e. the one with the smallest e-value) is listed. Click here for file

Additional file 8 **Schematic example of Newbler output.** Schematic example of contigs, isotigs, and isogroups produced by Newbler. Click here for file

Additional file 9 **Composition of the cDNA libraries in detail.** Two tables describe the cDNA libraries and the provenances in detail. Click here for file
