# Supplementary material for: A catalogue of putative unique transcripts from Douglas-fir (Pseudotsuga menziesii) based on 454 transcriptome sequencing of genetically diverse, drought stressed seedlings
Source: BMC Genomics. 2012 Nov 28;13:673. doi: 10.1186/1471-2164-13-673 (PMC3637476; doi:10.1186/1471-2164-13-673)
Supplement: Additional file 1 — Characteristics of the libraries. Number of reads and average read length per library before and after the pre-processing steps. [file 1471-2164-13-673-S1.pdf]

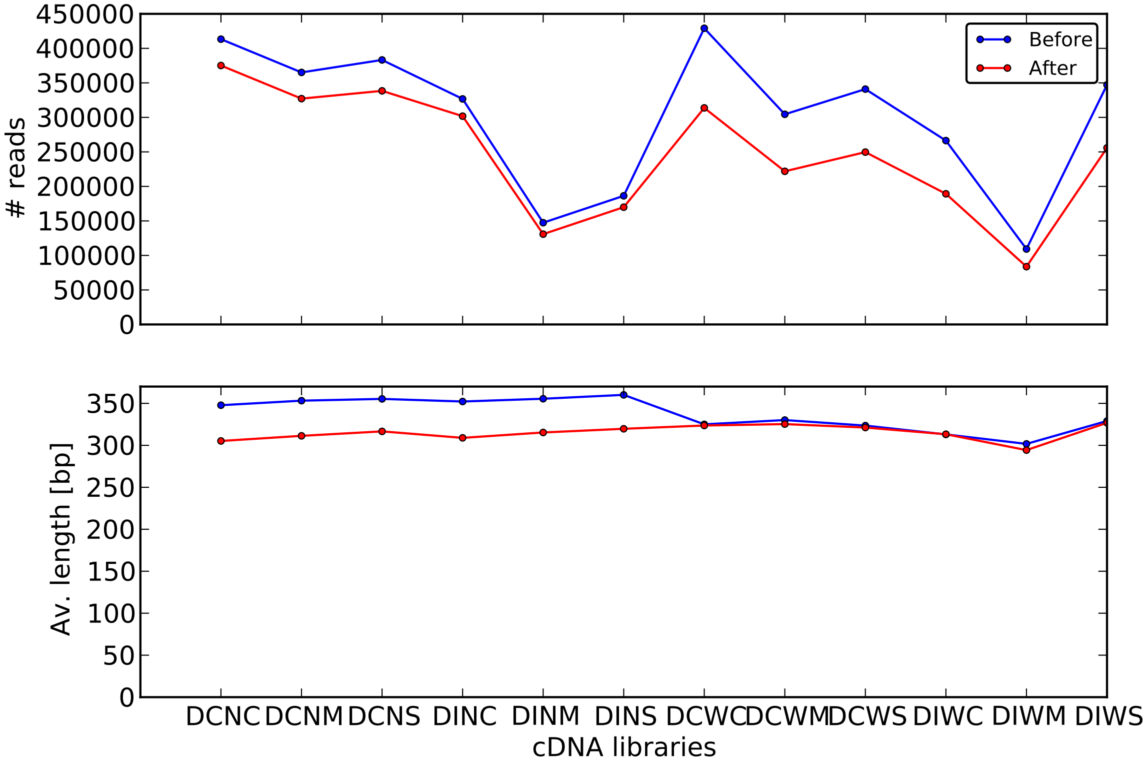

Additional Figure 1: Characteristics of the libraries. Number of reads and average read length per library before and after the pre-processing steps.
