# Supplementary material for: A catalogue of putative unique transcripts from Douglas-fir (Pseudotsuga menziesii) based on 454 transcriptome sequencing of genetically diverse, drought stressed seedlings
Source: BMC Genomics. 2012 Nov 28;13:673. doi: 10.1186/1471-2164-13-673 (PMC3637476; doi:10.1186/1471-2164-13-673)
Supplement: Additional file 2 — Read composition of the assembly. The origin as well as the number of reads assembled or otherwise marked by Newbler is illustrated. [file 1471-2164-13-673-S2.pdf]

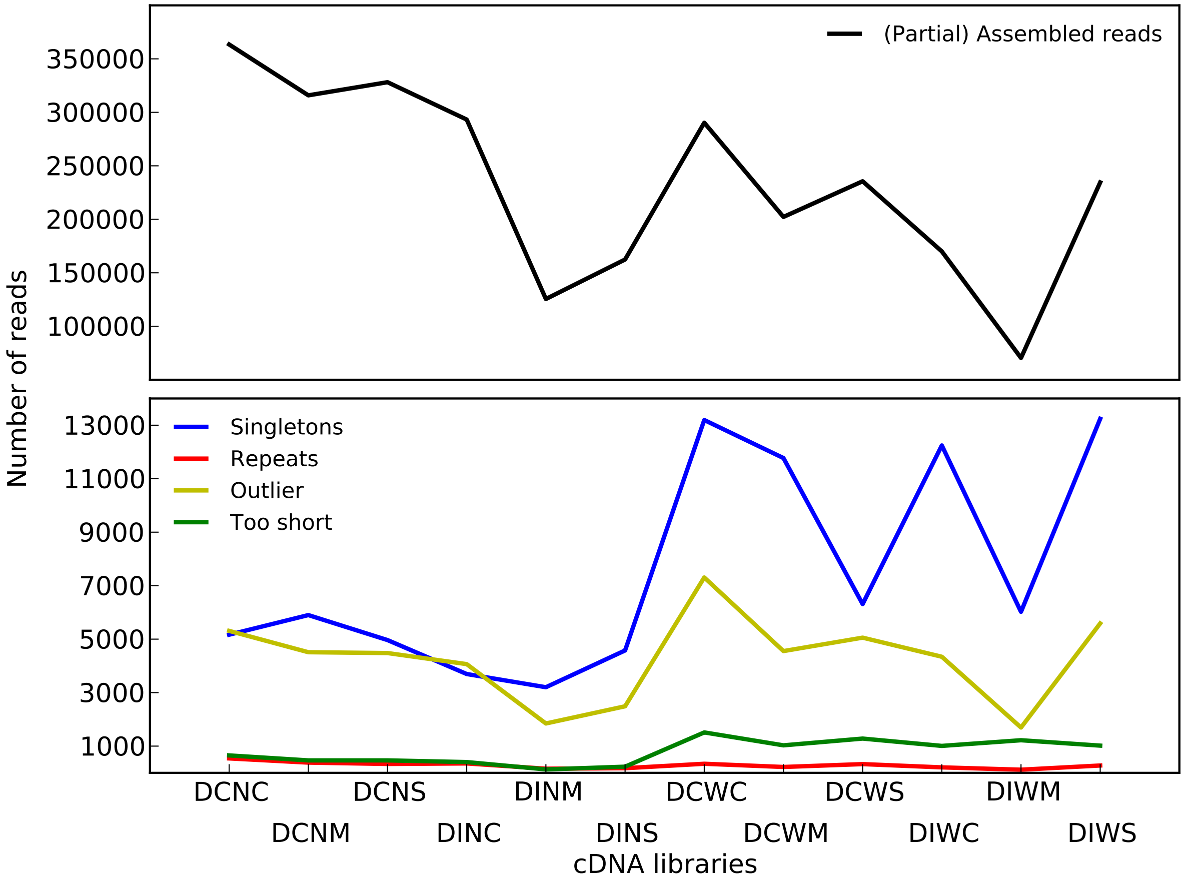

Additional Figure 2: Read composition of the assembly. The origin as well as the number of reads assembled or otherwise marked by Newbler is illustrated.
