# Supplementary material for: A catalogue of putative unique transcripts from Douglas-fir (Pseudotsuga menziesii) based on 454 transcriptome sequencing of genetically diverse, drought stressed seedlings
Source: BMC Genomics. 2012 Nov 28;13:673. doi: 10.1186/1471-2164-13-673 (PMC3637476; doi:10.1186/1471-2164-13-673)
Supplement: Additional file 3 — Log-log plot of assembled reads versus the sequence length. The log-log plot shows that the sequence length is depending on the number of reads assembled to the sequence. [file 1471-2164-13-673-S3.pdf]

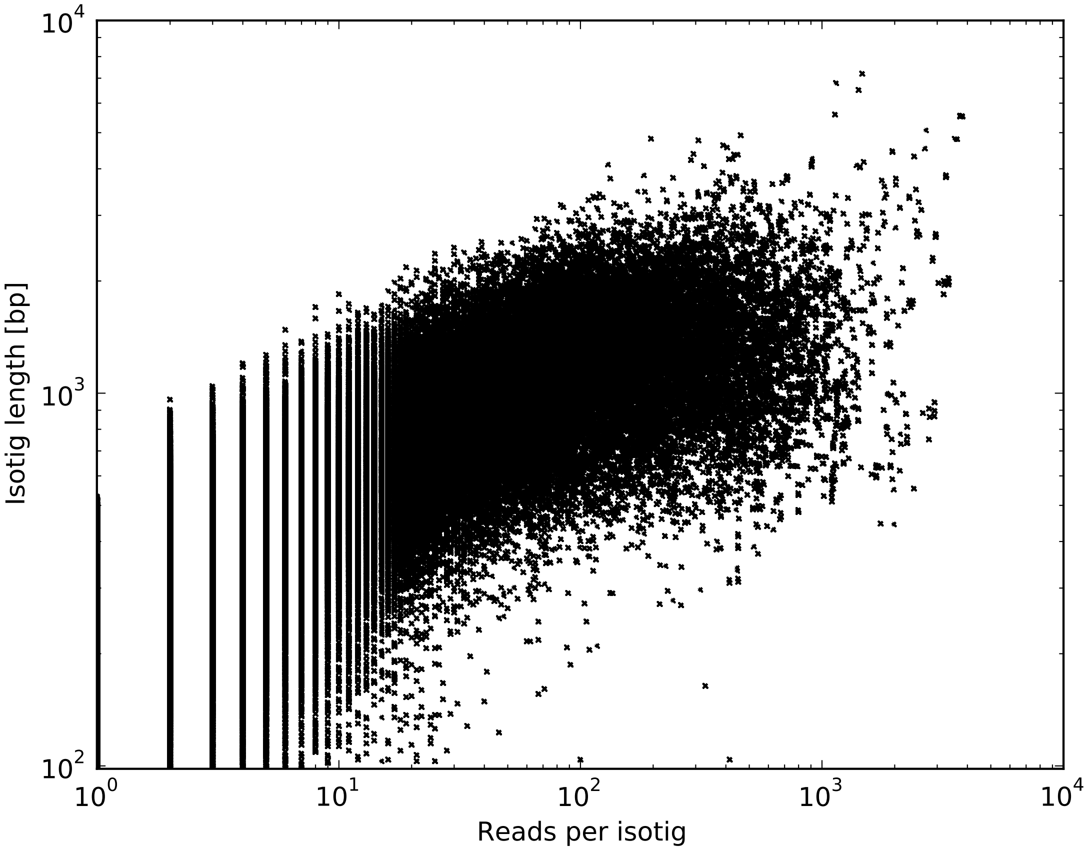

Additional Figure 3: The log-log plot shows that the sequence length is depending on the number of reads assembled to the sequence.
