# Supplementary figures and images for: A catalogue of putative unique transcripts from Douglas-fir (Pseudotsuga menziesii) based on 454 transcriptome sequencing of genetically diverse, drought stressed seedlings
Source: BMC Genomics. 2012 Nov 28;13:673. doi: 10.1186/1471-2164-13-673 (PMC3637476; doi:10.1186/1471-2164-13-673)

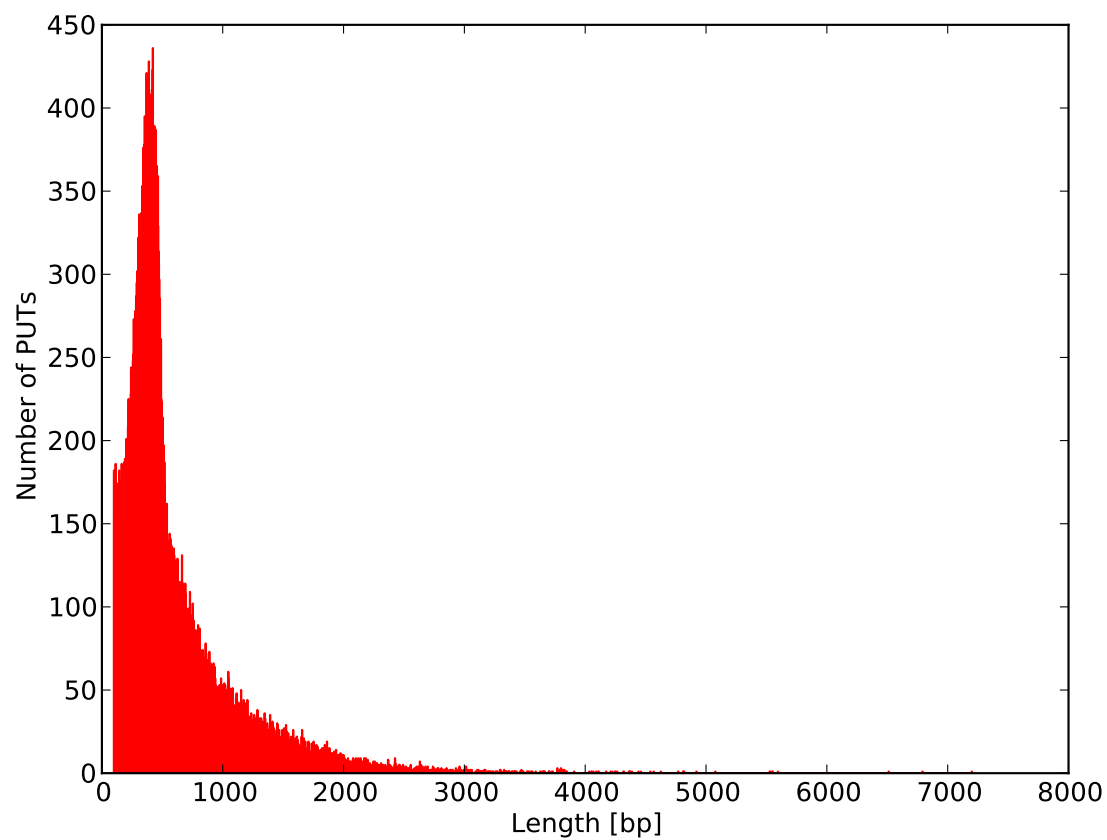

Additional Figure 4: Reads of all twelve cDNA libraries were assembled using Newbler.

Supplement: Additional file 4 — Number of isotigs per sequence length. Number of isotigs per sequence length. Reads of all twelve cDNA libraries were assembled using Newbler. [file 1471-2164-13-673-S4.pdf]
