# Supplementary material for: A catalogue of putative unique transcripts from Douglas-fir (Pseudotsuga menziesii) based on 454 transcriptome sequencing of genetically diverse, drought stressed seedlings
Source: BMC Genomics. 2012 Nov 28;13:673. doi: 10.1186/1471-2164-13-673 (PMC3637476; doi:10.1186/1471-2164-13-673)
Supplement: Additional file 5 — Comparison of the GO-Slim categories level 3 - 5. Comparison of the distribution of the GO-Slim categories of the Douglas-fir PUTs set versus Picea sitchensis and Arabidopsis thaliana at GO level 3 to 5. Transcriptome data of P. sitchensis and A. thaliana were obtained from NCBI and TAIR databases, respectively (See text for details). [file 1471-2164-13-673-S5.pdf]

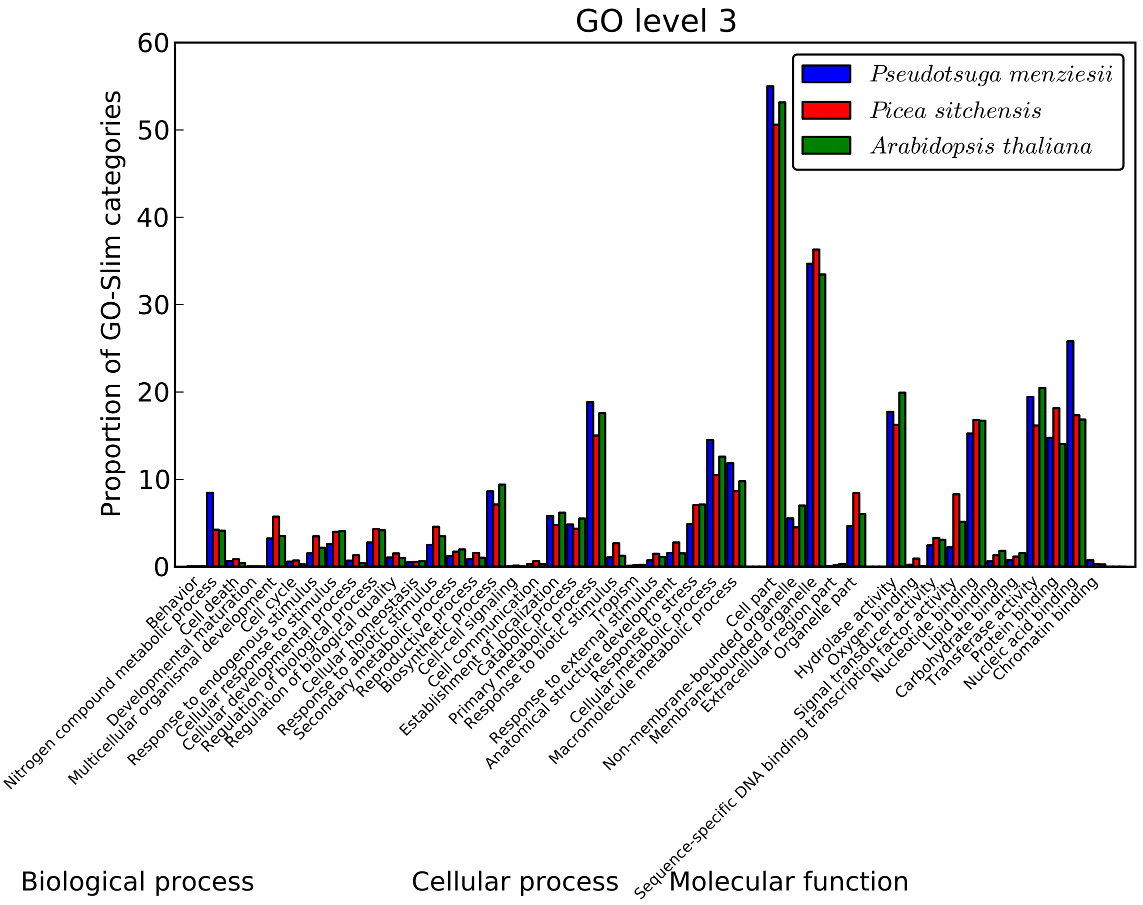

Additional Figure 5: Comparison of the distribution of the GO-Slim categories of the Douglas-fir PUTs set versus *Picea sitchensis* and *Arabidopsis thaliana* at GO level 3.

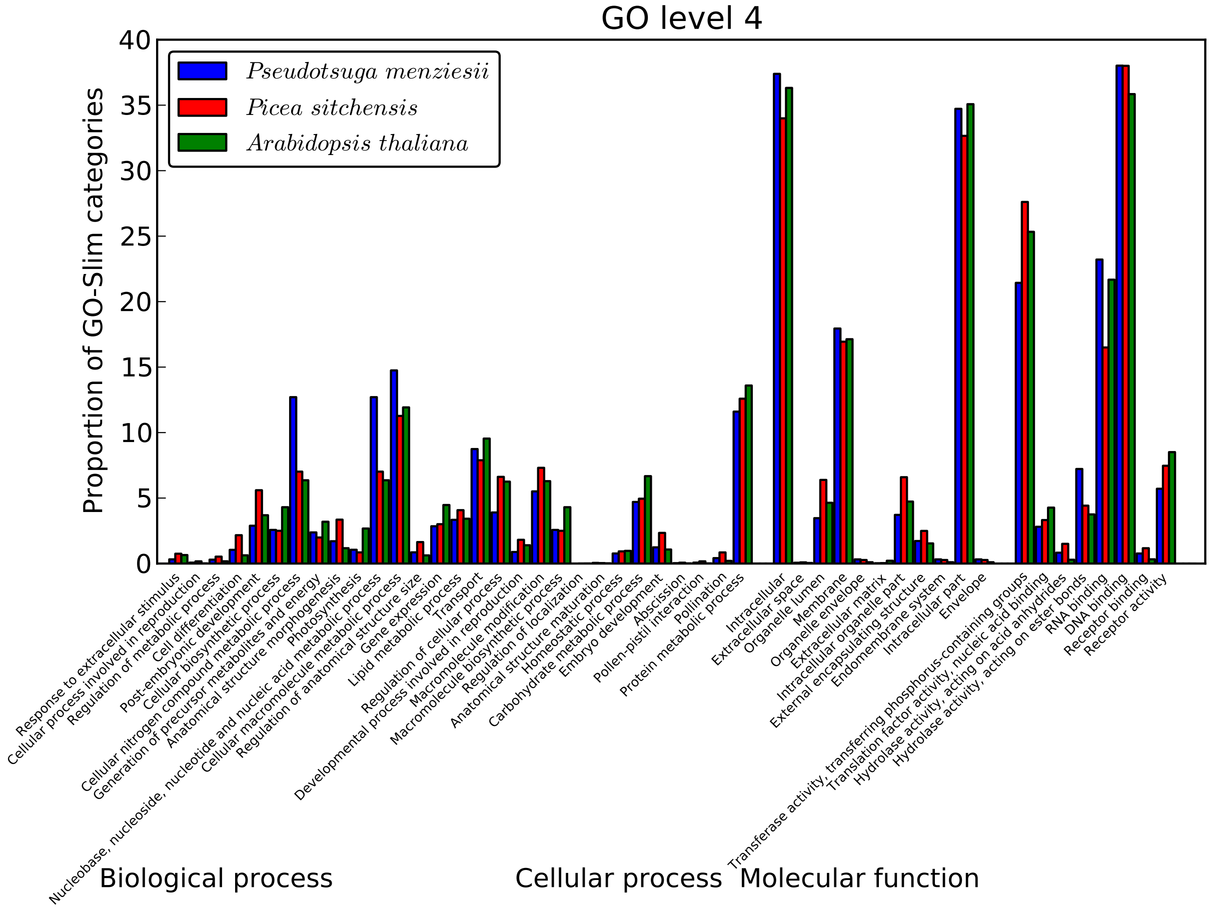

Additional Figure 6: Comparison of the distribution of the GO-Slim categories of the Douglas-fir PUTs set versus *Picea sitchensis* and *Arabidopsis thaliana* at GO level 4.

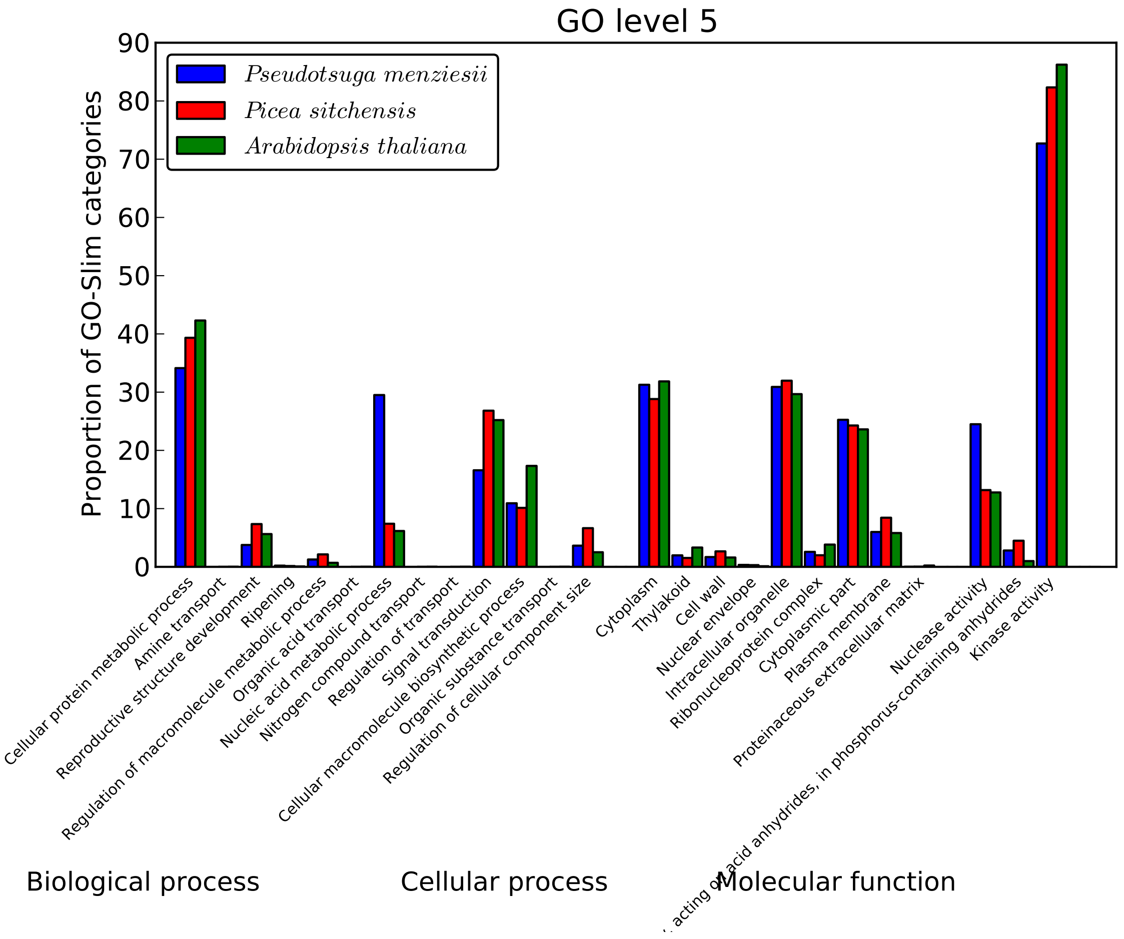

Additional Figure 7: Comparison of the distribution of the GO-Slim categories of the Douglas-fir PUTs set versus *Picea sitchensis* and *Arabidopsis thaliana* at GO level 5.
