# Supplementary material for: A catalogue of putative unique transcripts from Douglas-fir (Pseudotsuga menziesii) based on 454 transcriptome sequencing of genetically diverse, drought stressed seedlings
Source: BMC Genomics. 2012 Nov 28;13:673. doi: 10.1186/1471-2164-13-673 (PMC3637476; doi:10.1186/1471-2164-13-673)
Supplement: Additional file 6 — Number of identical BLASTX hits of different combination of groups after the keyword search. This table lists the number of identical BLASTX hits of different combination of groups after the keyword search. If combinations of sets are not listed, there were no equal BLASTX hits. [file 1471-2164-13-673-S6.pdf]

Additional Table 1: Number of identical BLASTX hits of different combination of groups after the keyword search.

Group(s)	Number of equal BLASTX hits
c cms cs ms	1
cm ms	1
cms cs ms s	1
c cm cms cs m s	1
cs m	1
c cm cms s	1
c cm cms m	1
c cms cs m s	1
c cm	1
cms cs ms	1
m s	1
cm cms s	1
cms cs m s	1
cm cms ms	1
c cms ms	1
c cm cms cs m ms s	1
c s	1
cm s	1
cms m s	2
c cms cs	2
c cm cms cs	2
c cm cms m ms s	2
c cm cms	2
cm cms m	2
m ms	2
c cms cs s	3
c cms s	3
cms cs m	3
cms ms s	4
cs s	4
ms	4
cm cms cs	5
cms cs s	8
cms m	9
cms ms	13
m	14
cm cms	17
cm	19
cs	20
s	21
c	24
cms s	27
cms cs	29
c cms	30
cms	557

Number of equal BLASTX hits (only results from the keyword search are considered) of isotigs (non-singleton PUTs) from different set(s) of groups (see Figure 3). In the not listed combinations of sets, there were no equal BLASTX hits. *c* = control, *m* = mild stress, *s* = severe stress, *cm* = control and mild stress, *cs* = control and severe stress, *ms* = mild and severe stress, *cms* = control, mild and severe stress.
