# Supplementary material for: A catalogue of putative unique transcripts from Douglas-fir (Pseudotsuga menziesii) based on 454 transcriptome sequencing of genetically diverse, drought stressed seedlings
Source: BMC Genomics. 2012 Nov 28;13:673. doi: 10.1186/1471-2164-13-673 (PMC3637476; doi:10.1186/1471-2164-13-673)
Supplement: Additional file 7 — BLASTX keyword search results. This file lists in a tab separated style for each BLASTX keyword search hit the following informations: keyword, isotig id, isotig group, hit id, hit definition, e-value. If there were more than one hit per keyword and isotig, only the best hit (i.e. the one with the smallest e-value) is listed. [file 1471-2164-13-673-S7.pdf]

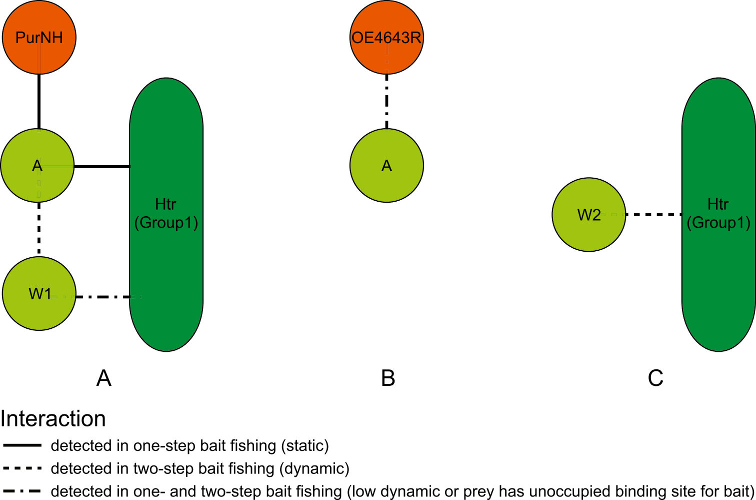
PurNH
A
W1
Htr
(Group1)
OE4643R
A
W2
Htr
(Group1)
A
B
C
Interaction
detected in one-step bait fishing (static)
detected in two-step bait fishing (dynamic)
detected in one- and two-step bait fishing (low dynamic or prey has unoccupied binding site for bait)
