# Supplementary material for: A catalogue of putative unique transcripts from Douglas-fir (Pseudotsuga menziesii) based on 454 transcriptome sequencing of genetically diverse, drought stressed seedlings
Source: BMC Genomics. 2012 Nov 28;13:673. doi: 10.1186/1471-2164-13-673 (PMC3637476; doi:10.1186/1471-2164-13-673)
Supplement: Additional file 8 — Schematic example of Newbler output. Schematic example of contigs, isotigs, and isogroups produced by Newbler. [file 1471-2164-13-673-S8.pdf]

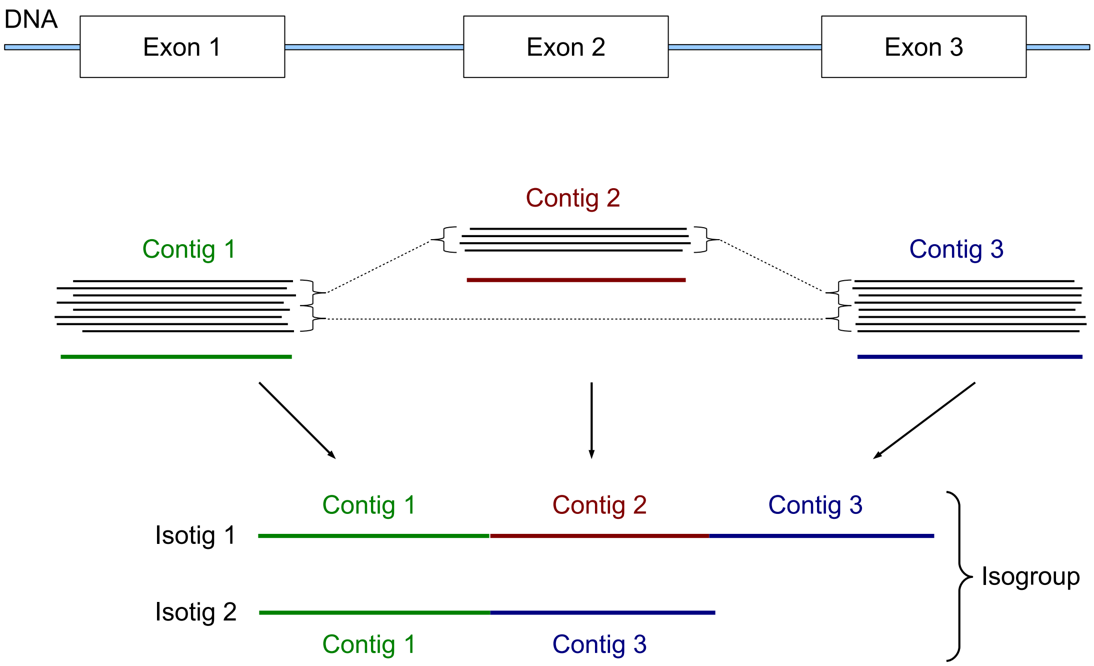

Additional Figure 8: Schematic example of contigs, isotigs, and isogroups produced by Newbler. Single reads (black lines) are assembled to contigs. The dotted lines represent subsets of reads implying connections between the contigs. The red, blue, and green line represent the consensus sequence of the contigs. The isogroup consists of two isotigs and in total three different contigs.
