# Supplementary material for: A catalogue of putative unique transcripts from Douglas-fir (Pseudotsuga menziesii) based on 454 transcriptome sequencing of genetically diverse, drought stressed seedlings
Source: BMC Genomics. 2012 Nov 28;13:673. doi: 10.1186/1471-2164-13-673 (PMC3637476; doi:10.1186/1471-2164-13-673)
Supplement: Additional file 9 — Composition of the cDNA libraries in detail. Two tables describe the cDNA libraries and the provenances in detail. [file 1471-2164-13-673-S9.pdf]

Additional Table 2: Composition of the cDNA libraries.

Library	Provenance	# individuals
DCNC	Lowry Lake	6
	Twin Harbors	6
	Pend Oreille	5
DCNM	Lowry Lake	5
	Twin Harbors	5
	Pend Oreille	4
DCNS	Lowry Lake	5
	Twin Harbors	5
	Pend Oreille	4
DINC	Twin Lake	6
	Salmon Arms	6
	Prince George	6
	Fort Collins	6
	Raton	6
DINM	Twin Lake	5
	Salmon Arms	5
	Prince George	4
	Fort Collins	4
	Raton	5
DINS	Twin Lake	4
	Salmon Arms	4
	Prince George	4
	Fort Collins	4
	Raton	5
DIWC	Twin Lake	7
	Salmon Arms	3
	Prince George	4
	Fort Collins	3
	Raton	3
DIWM	Twin Lake	4
	Salmon Arms	4
	Prince George	4
	Fort Collins	3
	Raton	5
DIWS	Twin Lake	3
	Salmon Arms	4
	Prince George	4
	Fort Collins	3
	Raton	3
DCWC	Lowry Lake	7
	Twin Harbors	5
	Pend Oreille	5
DCWM	Lowry Lake	6
	Twin Harbors	6
	Pend Oreille	4
DCWS	Lowry Lake	5
	Twin Harbors	5
	Pend Oreille	4

Additional Table 3: Origin of the provenances in detail.

Variety	Provenance	Country and province	Elevation	Climate	Experiment code	Nursery code
Costal	Lowry Lake	Canada, BC	185 m	very moist	BC2	FDC 1294
Costal	Twin Harbors	USA, WA	0-1000 m	very moist	WA1	FDC SP07-33
Costal	Pend Oreille	USA, WA	2800-3500 m	montane/dry	WA2	FDC PI06-144
Interior	Fort Collins	USA, CO	2500 m	montane/dry	CO	FDI 123
Interior	Raton	USA, NM	2300 m	montane/dry	NM	FDI unkown
Interior	Prince George	Canada, BC	850 m	Northern limit of range/dry	BC6	FDI 44913
Interior	Salmon Arms	Canada, BC	850 m	dry	BC3	FDI 39924
Interior	Twin Lake	Canada, BC	1067 m	very dry	BC1	FDI 2053
